# Organization of Interventional Neuroradiology in Belgium: results of a national survey

**DOI:** 10.1186/s13244-026-02416-z

**Published:** 2026-10-07

**Authors:** Adam Dmytriw, Iman Kiani, Luciana Koren, Hamza Salim, Thomas Lefebvre, Matea Prenc, Chiara Brechbühl, Denis Brisbois, Frans Vanden Bergh, Maurits Voormolen, Stephanie Elens, Luca Scarcia, Vivek Yedavalli, Louis Deprez, Yacine Boudiba, Adrien Guenego

**Affiliations:** 1 Neurointerventional & Neuroanalytics Consortium (NAN-C), School of Medicine, Toronto Metropolitan University, Toronto, ON Canada; 2 Nuffield Department of Surgical Sciences, Faculty of Clinical Medicine, University of Oxford, Oxford, UK; 3 Students’ Scientific Research Center, Tehran University of Medical Sciences, Tehran, Iran; 4 School of Medicine, University of Zagreb, Zagreb, Croatia; 5 Department of Radiology, Division of Neuroradiology, Johns Hopkins Medical Center, Baltimore, MD USA; 6 Department of Neuroradiology, MD Anderson Medical Center, Houston, TX USA; 7 Department of Interventional neuroradiology, CUB Hôpital Erasme, Université libre de Bruxelles (ULB), Brussels, Belgium; 8 Department of Diagnostic and Interventional Radiology, University Hospital Center Sestre milosrdnice, Zagreb, Croatia; 9 Faculty of Medicine, Department of Neurology, Inselspital, Bern University Hospital, University of Bern, Bern, Switzerland; 10 CH Liege, Liege, Belgium; 11 VUB, Brussels, Belgium; 12 CH, Antwerp, Belgium; 13 CH Charleroi, Charleroi, Belgium; 14 Department of Neuroradiology, Hôpitaux Universitaires Henri Mondor, Créteil, France; 15 Division of Neuroradiology, Department of Radiology, Johns Hopkins Medical Center, Baltimore, MD USA; 16 Department of Diagnostic and Interventional Neuroradiology, CH Liege, Liege, Belgium

**Keywords:** Interventional neuroradiology, Mechanical thrombectomy, Workforce organization, On-call systems, Training pathways

## Abstract

**Objectives:**

To provide a comprehensive national overview of interventional neuroradiology (INR) in Belgium through a structured survey conducted under the auspices of the Belgian Society for Interventional and Therapeutic Neuroradiology (BSITN).

**Materials and methods:**

An anonymized online survey was distributed by email through the BSITN to all known active interventional neuroradiologists. The questionnaire assessed workforce demographics, organization of INR services, procedural activity, on-call systems, training and certification pathways, and remuneration models. Associations were tested using Fisher’s exact or chi-square tests. Workforce projections were estimated using a stock-flow model based on respondent age distribution and current training pipeline capacity.

**Results:**

Thirty-five interventional neuroradiologists responded, representing more than 70% of the estimated active INR workforce in Belgium. Respondents were predominantly senior physicians (88.6%), with nearly half reporting more than 10 years of post-fellowship practice. Annual center-level procedural volumes were high, exceeding 200 cases in two-thirds of responses. Despite this variability, 68.6% of respondents felt sufficiently exposed across the full spectrum of INR procedures. Greater professional experience (> 10 years) was strongly associated with perceived procedural sufficiency (*p* = 0.001). A 24/7 stroke unit was available for 33 of 35 physicians. Workforce projections indicated a potential decline of approximately 23% over the next decade under current training capacity.

**Conclusions:**

This national BSITN survey reveals significant variability in Belgian INR organizational models. The results of the survey also point to challenges related to workforce sustainability, training standardization, and service organization. As such, they can serve as a reference point for coordinated national initiatives.

**Key points:**

**Question** While procedural efficacy of INR is well established, the organizational structures, workforce characteristics, and training pathways that underpin INR services have not been systematically described at a national level.

**Findings** This study showed significant variability in institutional models, on-call systems, training pathways, and remuneration across centers. While a 24/7 stroke unit was available for 33 of 35 physicians, workforce projections indicated a potential 23% decline in the active INR workforce by 2035 under current training capacity.

**Critical relevance statement:**

These data establish the first national organizational baseline for Belgian INR and identify an impending workforce shortfall that threatens the sustainability of time-critical neurovascular services. The strong professional consensus favoring a standardized national training curriculum provides a clear mandate.

**Graphical Abstract:**

**Figure:**
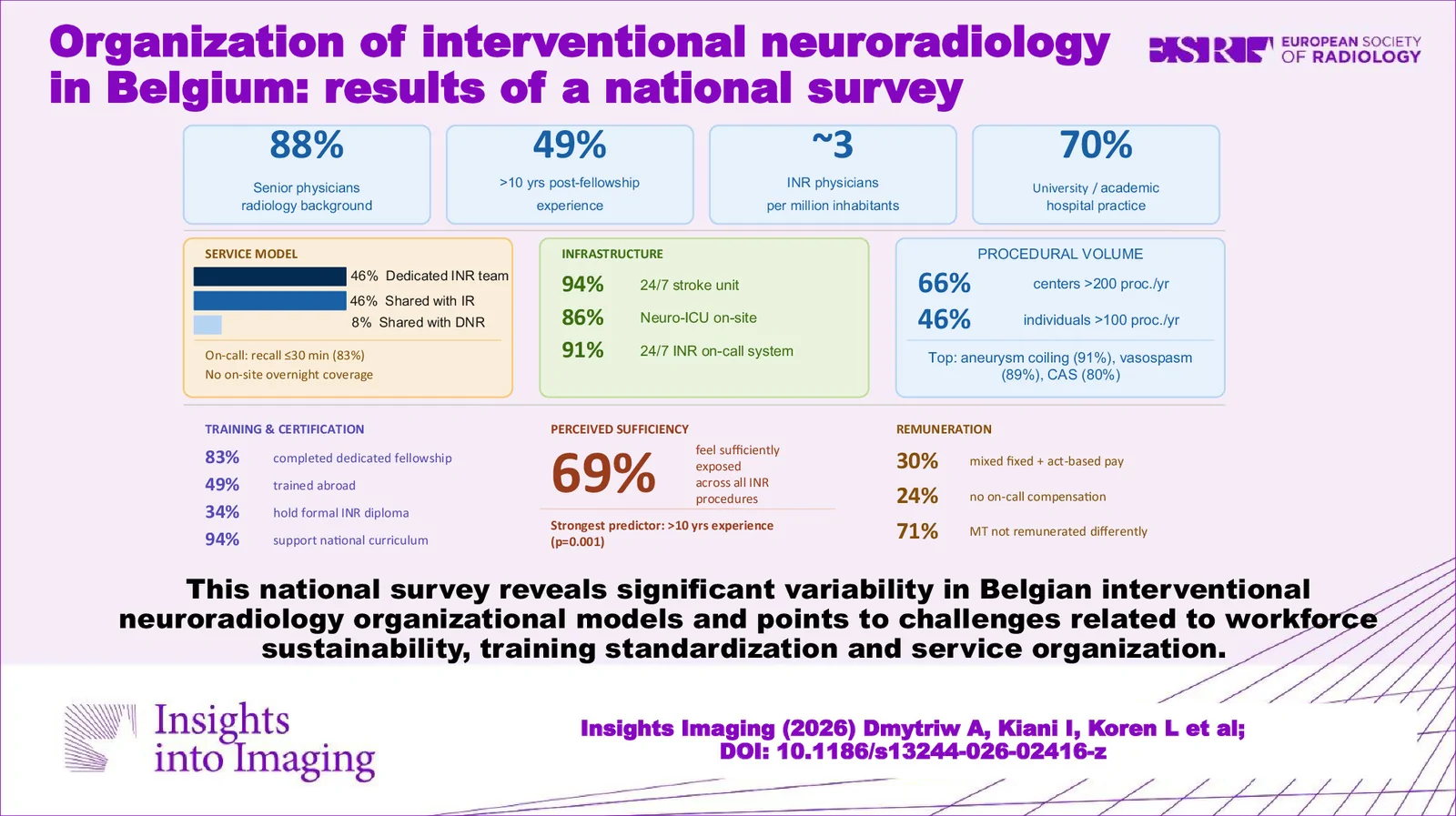

## Introduction

Interventional neuroradiology (INR) has become an essential diagnostic and therapeutic option for managing vascular conditions in the brain, head, neck, and spine. It provides minimally invasive treatment options for complex pathologies, such as intracranial aneurysms, vascular malformations, acute ischemic stroke, chronic subdural hematomas (cSDHs), pulsatile tinnitus, and selected carotid stenosis [1]. Over the past decade, the role of INR has expanded considerably, especially following the widespread adoption of mechanical thrombectomy as standard treatment for acute ischemic stroke due to large vessel occlusion [2, 3]. As a result, INR services are now expected to provide continuous availability and deliver time-critical interventions while ensuring functionality within highly coordinated multidisciplinary systems.

Although the clinical effectiveness of INR procedures is well established in recent literature, less attention has been devoted to the organizational, workforce, and training structures required to maintain these services safely and sustainably [4]. Factors such as appropriate infrastructure, resilient staffing models, standardized training pathways, and sustainable on-call systems have been identified as important contributors in maintaining procedural quality and patient safety [5, 6]. The requirement for 24/7 availability, frequent on-call duties, and high-intensity emergency work, places significant and ongoing demands on a relatively small and highly specialized workforce, raising concerns regarding long-term retention and succession planning [7–9].

In Belgium, INR services have developed progressively within a dense hospital network, alongside the widespread implementation of mechanical thrombectomy. However, to date, no national data have been published describing how INR is organized at the physician level, from workforce composition and institutional models to training pathways, on-call organization, and remuneration. The Belgian Society for Interventional and Therapeutic Neuroradiology (BSITN), which brings together the majority of practicing interventional neuroradiologists in Belgium, is uniquely positioned to address this knowledge gap. The aim of the present study was therefore to provide a comprehensive national overview of the organization of INR in Belgium through a structured survey conducted under the auspices of the BSITN.

## Materials and methods

### Study design

This study was a national anonymized online survey conducted under the auspices of the BSITN. The questionnaire was distributed to all active interventional neuroradiologists in Belgium, including residents, fellows, and senior staff. At the time of the survey, fewer than 50 physicians were identified as being actively involved in INR practice nationwide and constituted the targeted population of the study.

### Survey development and data collection

The questionnaire was developed by a BSITN board member and actual President (A.G.). An invitation email was distributed by the BSITN to its members, who represent the national community of physicians actively involved in INR practice in Belgium, and non-members involved in INR practice in Belgium. The email included information regarding the purpose of the study, the voluntary nature of participation, the approximate time required to complete the survey, and a direct link to the questionnaire. The questionnaire was designed to evaluate the organization of INR practice in Belgium and covered the following domains: professional background, institutional organization, scope of neurointerventional activity, on-call systems and workload, training pathways and certification, and remuneration models. The survey consisted predominantly of single-choice and multiple-choice questions, with a limited number of open-ended questions to allow additional comments. Survey questions were non-randomized and presented in a fixed order. In accordance with standard survey methodology, questions related to compensation were placed toward the end of the questionnaire. Respondents were able to review and modify their answers prior to final submission. Participation was voluntary, and no compensation was offered. Data collection took place between 23 November and 15 December 2025. To reduce the risk of duplicate submissions, the survey was configured to allow only one completed response per IP address. Responses were collected anonymously, and no directly identifying personal data were requested or stored.

### Ethics approval disclosure

The survey collected anonymous data from physicians only and did not involve patients, patient-level clinical information, or clinical interventions. In accordance with local practice for anonymized professional surveys, formal ethics committee approval was not needed. Completion of the survey was considered an implication of informed consent to participate.

### Data collection and statistical analysis

No imputation of missing data was performed. Categorical variables were reported as absolute numbers and percentages, with percentages calculated using the number of respondents for each individual question as the denominator. Associations between categorical variables were assessed using Fisher’s exact test for 2 × 2 tables and the chi-square test for larger contingency tables. A *p*-value of < 0.05 was considered statistically significant.

Workforce projections were estimated using a stock-flow model based on the age distribution of respondents. Near-term attrition was defined as respondents aged 60 years or older, assumed to retire within 5 years. Medium-term attrition included respondents aged 50–59 years, projected to retire within 5–15 years. The training pipeline was calculated as the sum of current residents, fellows, and practitioners with fellowship training in progress or planned. The projected workforce gap was estimated as the difference between anticipated retirements and the training pipeline.

All analyses were performed using Python 3.10 with pandas, scipy, and matplotlib libraries.

## Results

### Respondent characteristics

A total of 35 interventional neuroradiologists responded to the survey, corresponding to more than 70% of the estimated active INR workforce in Belgium. Respondents were male in 32/35 cases (91.4%), and 31/35 (88.6%) were senior physicians, all with a radiology background: only 2/35 (5.7%) were residents and 1/35 (2.9%) was a fellow. The 12/35 (34.3%) were between 30 and 39 years of age, followed by those aged 40–49 years (9/35, 25.7%) and 50–59 years (9/35, 25.7%) (Table 1).

**Table 1:** Demographic and professional characteristics of survey respondents

Characteristic	*n* (%)
Sex, male	32 (91.4)
Age 30–50 years	21 (60.0)
Senior physician	31 (88.6)
> 10 years of INR experience	17 (48.6)
Practice in a university hospital	24 (70.0)
Practice in ≥ 2 hospitals	11 (31.4)

Data are presented as absolute numbers (*n*) and percentages (%); percentages are calculated using the number of respondents for each item as the denominator. University practice is defined as respondents working in academic hospital networks or mixed university-non-university settings

### Practice setting, multi-center activity and procedural volumes

Annual center volumes were > 200 procedures in 23/35 (65.7%) and 100–200 in 11/35 (31.4%). 1/35 (2.9%) reported a center volume of 51–99 procedures, and no respondent reported fewer than 50 procedures annually (Supplementary Table S1). As the survey was administered at the individual physician level, the 35 respondents represented 20 distinct institutions.

Diagnostic angiography was performed by 34/35 respondents (97.1%), mechanical thrombectomy by 33/35 (94.3%), aneurysm coiling/remodeling by 32/35 (91.4%), and vasospasm treatment by 31/35 (88.6%). At the team level, annual volumes exceeding 100 cases/year were reported for mechanical thrombectomy by 22/35 respondents (62.9%) and for aneurysm embolization by 14/35 (40.0%). Annual team volumes of fewer than 10 cases/year were reported for venous stenting by 21/35 respondents (60.0%) and for cSDH embolization by 15/35 (42.9%) (Supplementary Fig. S1).

### Organization of INR services and on-call workload

INR services were organized as a dedicated INR team in 16 of 35 cases (45.7%). An equivalent proportion of respondents (16/35, 45.7%) indicated that INR activity was shared with general interventional radiology, while 3 of 35 (8.6%) reported activity shared with diagnostic neuroradiology. A 24/7 stroke unit was available for 33 of 35 physicians (94.3%) and on-site neuro–intensive care for 30 of 35 physicians (85.7%).

Seventeen of thirty-five (48.6%) respondents reported having dedicated neurointerventional radiographers/technicians, and 15 of 35 (42.9%) reported dedicated neurointerventional nursing staff. In the remaining responses, radiographers and nursing staff were shared with other interventional or radiology activities. A 24/7 INR on-call system was reported by 32/35 respondents (91.4%). Immediate recall (typically < 30 min) was reported by 29/35 (82.9%) and recall with response times longer than 30 min by 6/35 (17.1%); no respondent reported on-site coverage. Ten of thirty-five respondents (28.6%) participated in a shared on-call system with another hospital, while 20 of 35 (57.1%) reported no shared coverage. Five respondents (14.3%) indicated that shared on-call arrangements were being considered as a potential future solution.

### Training pathways, certification and remuneration

**Fig. 1:**
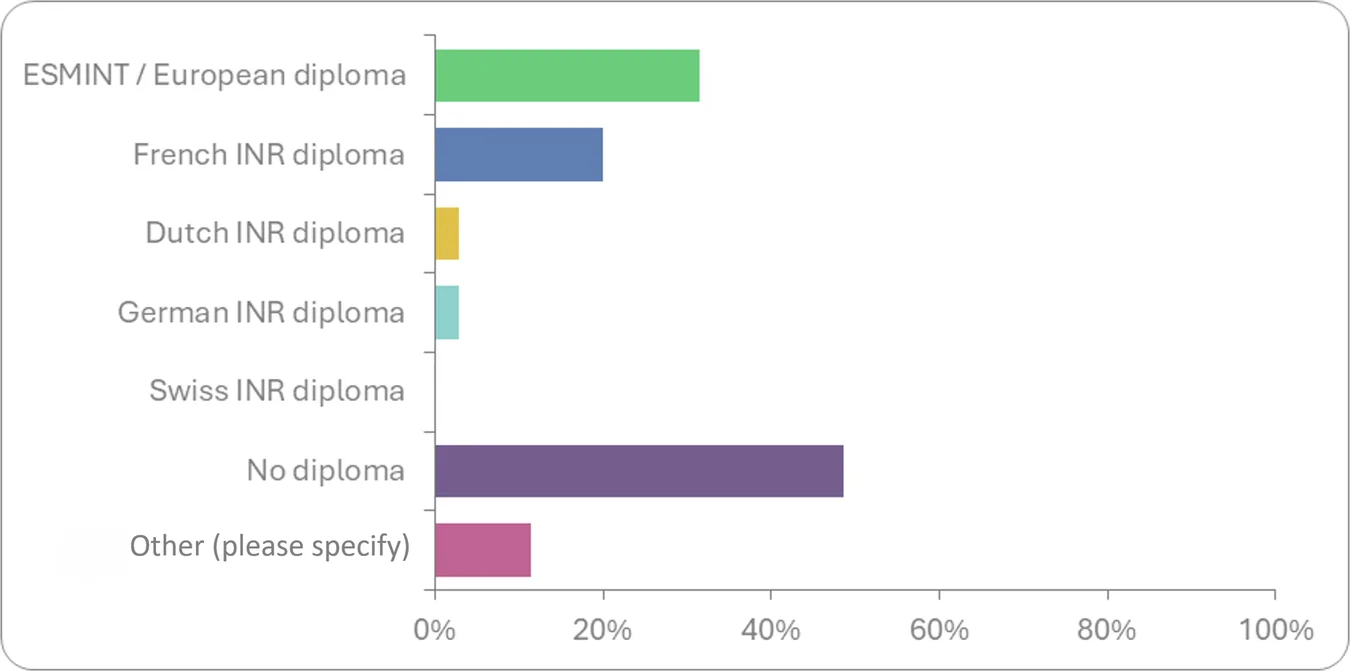
Distribution of responses to the survey question: “Do you hold an official or theoretical INR diploma or certification? If yes, which theoretical diploma(s)?” ESMINT, European Society of Minimally Invasive Neurological Therapy; INR, interventional neuroradiology

A dedicated INR clinical fellowship was completed by 29/35 respondents (82.9%), of whom 17/35 (48.6%) completed ≥ 2 years. Training location was Belgium in 12/35 (34.3%) and abroad in 17/35 (48.6%) (Supplementary Table S2). Training was limited to workshops or short courses in 3/35 (8.6%) and to observerships in 2/35 (5.7%). One respondent (1/35, 2.9%) completed no formal INR training. A formal theoretical or official INR diploma was held by 12/35 (34.3%), while 4/35 (11.4%) reported certification in progress and 19/35 (54.3%) reported no diploma (Fig. 1).

Remuneration consisted of a combined fixed and act-based payment in 10/33 (30.3%), fixed allowance only in 8/33 (24.2%), and fee-per-procedure only in 5/33 (15.2%). No specific on-call compensation was present in 8 of 33 cases (24.2%). Mechanical thrombectomy was not remunerated differently from elective neurointerventional procedures in 22 of 31 cases (71.0%); two additional respondents omitted this specific item on thrombectomy reimbursement, reducing the denominator from 33 to 31.

Support for a standardized national INR training curriculum was reported by 33/35 (94.3%); 34/35 (97.1%) supported a BSITN/BSR-hosted national list of INR-trained physicians.

### Factors associated with perceived volume sufficiency

The 24/35 respondents (68.6%) reported feeling sufficiently exposed across the full spectrum of neurointerventional procedures. Among respondents reporting sufficient exposure, 67% had > 10 years of INR experience vs 8% among those reporting insufficient exposure (*p* = 0.001). University-based practitioners showed a trend toward lower satisfaction (29% vs 67%, *p* = 0.07). Other factors including personal volume, fellowship completion, diploma status, and availability of dedicated staff were not significantly associated with perceived sufficiency (Supplementary Table S3).

### Workforce projections

**Fig. 2:**
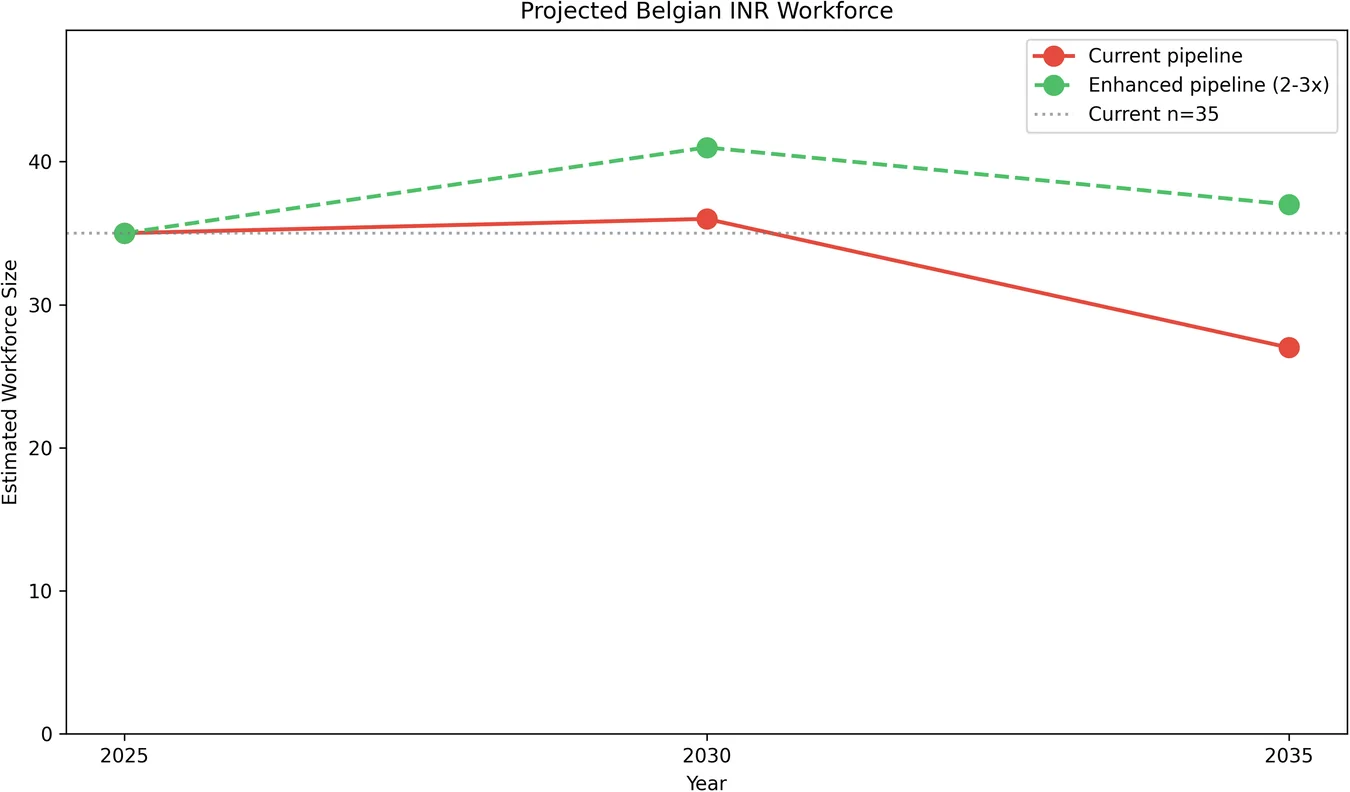
Projected Belgian INR workforce from 2025 to 2035. Projections are based on anticipated retirements (respondents aged ≥ 50 years) and current training pipeline (residents, fellows, and fellowships in progress). The red line represents workforce trajectory under current training capacity. The green dashed line represents an enhanced scenario with 2–3 times the current fellowship output. The gray dotted line indicates the current workforce size (*n* = 35)

Based on the age distribution of respondents, an estimated 4 practitioners (11%) were aged 60 years or older and 9 (26%) were aged 50–59, suggesting potential attrition of 13 practitioners (37%) over the next 10–15 years. The current training pipeline comprised 5 individuals (2 residents, 1 fellow, 2 with fellowships in progress). Under current training capacity, the projected workforce would decline from 35 to approximately 27 by 2035. Doubling or tripling fellowship positions could maintain or modestly increase the workforce to 37 practitioners over the same period (Fig. 2).

## Discussion

INR is a highly specialized, yet small field that continues to shape modern neurovascular practice. Furthermore, it has become a central component in the management of cerebrovascular disease, combining minimally invasive techniques with proven clinical benefit [10]. Given its impact on both patient outcomes and healthcare resource utilization, understanding how INR services are organized in real-world settings and how responsibilities, training, and professional recognition are structured, has become essential for ensuring quality, safety, and long-term development.

The demographic profile of respondents provides important context for interpreting the organizational findings. The Belgian INR workforce appears small and predominantly composed of senior physicians, reflecting a field largely driven by experienced practitioners. Nevertheless, it is worth mentioning that across Europe, growth in thrombectomy- capable centers has not always been accompanied by proportional expansion of training capacity or recruitment of younger practitioners [11, 12].

Another interesting finding of this survey is the variability in how INR is organized across Belgian centers. Although nearly all respondents reported access to core neurovascular infrastructure, such as 24/7 stroke units and neuro–intensive care in most cases, integration of INR activity and internal staffing models differ considerably between institutions. These differences are of great interest and clinical relevance, as organizational integration may influence the availability of dedicated personnel, team familiarity, and consequently workflow efficiency [13]. Similar patterns have been described in other European settings. Data from the Stroke Action Plan for Europe’s 2023 service tracker show persistent differences in organization, delivery, and equity of stroke care across countries, regardless of overall improvements in treatment access [14].

When placed in an international context, the Belgian INR workforce density appears broadly comparable to that of other Western European countries, though such comparisons must account for differences in specialty definitions and practice structures. In Belgium, fewer than 50 active INR physicians serve a population of approximately 11.6 million, yielding a density of roughly three practitioners per million inhabitants. In early 2023, France reported 183 tenured INR professionals, which for a population of approximately 68 million translates to a comparable ratio of around 2.7 physicians per million [15]. Similarly, recent survey-based studies have documented variation in thrombectomy practices and ongoing staffing challenges across European centers [16]. At a global level, variability in access to reperfusion therapies further shows the absence of unified organizational models [17]. The organization of thrombectomy-capable centers differs considerably between countries. France operates approximately 38–56 MT centers across a population of around 68 million [15], while Germany, with a population of around 84 million, has developed approximately 200 MT centers and 332 stroke units [18], reflecting a far more decentralized model of care delivery. The German model, with nearly five times more centers relative to population compared to France, reflects a deliberate policy choice to maximize geographic coverage and reduce transfer delays, at the cost of lower individual center volumes. Belgium demonstrates predominantly high-volume centers, with two-thirds exceeding 200 procedures annually but with fewer exceeding 100 mechanical thrombectomy procedures per year, suggesting a more atypical model between the French and German paradigms. Whether this concentration reflects deliberate planning or an emergent consequence of a small national workforce remains a question this survey alone cannot fully resolve, and represents a topic deserving of further policy attention. Beyond these national examples, directly comparable physician-level organizational data remain scarce, as most international literature has focused on procedural workflow and treatment access rather than on workforce structure, on-call organization, training pathways and remuneration. The pan-European ESMINT survey of mechanical thrombectomy practices similarly highlighted wide variation in service organization across centers [13], and consensus guidance from the United Kingdom has underscored the staffing and on-call requirements needed to deliver thrombectomy safely [5]. The consistency of these themes across settings suggests that the organizational and workforce pressures identified in Belgium are not country-specific but reflect broader challenges of sustaining a small, highly specialized workforce, and that internationally harmonized data collection would strengthen future cross-national comparisons.

An additional organizational dimension recognized by the survey is the structure of on-call systems. In Belgium, continuous INR coverage predominantly relies on recall-based on-call systems supported by small teams, often without shared coverage between institutions, although some of the respondents identified the interinstitutional on-call system as an interesting solution. Although the present survey did not directly assess practitioner well-being, previous studies in neurointerventional practice and interventional radiology have linked intensive call schedules to sleep deprivation, burnout and self-reported medical errors, raising concerns about both practitioner well-being and patient safety [19]. Comparable trends have been documented in interventional radiology and other procedural specialties, suggesting that these pressures are not unique to INR and reflect broader systemic challenges associated with delivering 24/7 specialist care [20]. Notably, while only a minority reported considering shared on-call cover, this may represent a potential strategy to reduce on-call strain and should be explored in future service planning.

Furthermore, the results of the survey identify training pathways and formal certification as another important source of variability, while simultaneously revealing a clear national consensus on how these issues should be addressed. Although the majority of respondents completed dedicated INR fellowships, training duration, supervision, and certification varied. In terms of the existence of clearly defined training pathways and formal certification, only a minority had completed an official theoretical diploma. Conversely, almost all respondents expressed support for a standardized national INR training curriculum and the creation of a BSITN-hosted national list of INR-trained physicians. It should, however, be noted that according to the European Society of Minimally Invasive Neurological Therapy, passing the training course is considered a requirement [21].

The final aspect addressed by the survey concerned remuneration. In most cases, on-call activity was compensated by the employing institution through a mixed model combining fixed allowances and act-based payments. However, one must note that nearly one quarter of respondents stated that they receive no specific compensation for on-call duties. In addition, mechanical thrombectomy was not remunerated differently from elective neurointerventional procedures or aneurysm treatment in the majority of cases. When placed in the broader context of health systems research across procedural specialties, such findings emphasize that misalignment between workload, responsibility and remuneration may have implications on job satisfaction, recruitment, retention and service continuity [22].

The finding that experience, rather than actual procedural volume, was the strongest predictor of perceived sufficiency is noteworthy. Early-career neurointerventionalists appear to feel under-exposed regardless of their objective caseload, suggesting that confidence in procedural competence accumulates gradually over years of practice. This has implications for training and mentorship programs, which may need to extend structured supervision and feedback beyond the fellowship period. Similar patterns have been observed in other procedural specialties, where self-assessed competence often lags behind objective metrics during the early independent practice phase [23]. The paradoxical trend toward lower satisfaction among university-based practitioners despite generally higher institutional volumes warrants consideration. In academic centers, cases are often distributed among larger teams comprising multiple consultants, fellows, and trainees, potentially diluting individual exposure [24]. It should also be noted that lower procedural volume, as one respondent indicated (fewer than 20 procedures per year), may increase the risk of complications [25].

Lastly, our workforce projections highlight a potential sustainability challenge for Belgian neurointerventional services. With over one-third of current practitioners approaching retirement age and a limited training pipeline, maintaining the current workforce will require deliberate expansion of fellowship capacity. The projected decline from 35 to 27 practitioners by 2035 under current conditions would occur against a backdrop of increasing demand driven by population aging and expanding indications for mechanical thrombectomy. Notably, in line with the European Training Requirements in INR of the UEMS Division of Neuroradiology, a dedicated INR fellowship typically lasts two years, which was also the most common duration reported by respondents (17/35 completed two years or more) [21]. However, it should also be noted that Belgium has no legal restrictions on fellowship duration. Overall, these projections are inherently simplified and do not account for factors such as immigration, early retirement, or changes in practice patterns, but they underscore the need for proactive workforce planning at the national level with the BSITN.

Several directions for future research emerge from these findings. First, while this survey identifies several structural occupational stressors, including 24/7 on-call coverage with stringent response-time requirements and an absence of dedicated on-call compensation in nearly a quarter of respondents, it did not directly assess practitioner well-being. Subsequent studies should employ validated psychometric instruments such as the Maslach Burnout Inventory to quantify burnout prevalence and examine its relationship with specific organizational models. Second, the centralized structure of Belgian INR services, with the majority of activity concentrated in a small number of high-volume centers, raises questions about equitable access for patients in peripheral or rural regions. Future work should cross-reference these organizational data with geospatial mapping of center locations and stroke transfer pathways to determine whether the Belgian model introduces disparities in door-to-groin times compared to more decentralized systems such as Germany’s. Third, the current survey was limited to physicians; yet INR services are critically dependent on specialized support personnel, and more than half of respondents indicated that radiographers and nursing staff are shared with general interventional radiology departments. Broadening the survey framework to include neurointerventional nurses and radiographers would provide a more complete picture of operational vulnerabilities and inform workforce planning beyond the physician tier.

## Limitations

This study has limitations inherent to its survey-based design. Firstly, all data were self-reported and may therefore be subject to reporting or recall bias. In addition, because the survey was administered at the individual physician level, several institutions were represented by more than one respondent; the 35 respondents corresponded to approximately 20 distinct centers. Center-level metrics such as procedural volume therefore reflect physician-reported activity and may include overlapping or duplicated institutional data, and should be interpreted accordingly. Moreover, because institutional affiliation was captured as a free-text field, a small number of responses were ambiguous or could not be unequivocally attributed to a specific center, such that the number of distinct institutions represented could only be approximated. Secondly, the survey did not include independent verification of institutional characteristics, procedural volumes, or on-call arrangements. Thirdly, although the response rate of the survey exceeded 70%, non-respondents opinions and characteristics may differ from respondents, which could introduce selection bias. In addition, the survey captures practices at a single time point, thereby not accounting for recent or ongoing organizational changes within institutions. However, the high response rate relative to the estimated national INR workforce and the broad representation across centers provide a meaningful overview of current INR practice in Belgium. Moreover, a prospective national database with government funding, including all patients treated with INR, could help provide a better vision and planning.

## Conclusions

This national BSITN-led survey provides the first comprehensive overview of how INR is organized in Belgium, focusing on both the common patterns and important discrepancies in organizational structures, training pathways, workload distribution, and remuneration models. Beyond its descriptive value, this work represents an essential first step toward a more integrated and forward-looking approach to the organization of INR services at a national level. With attention to concrete differences in organizational models, we sought to provide a basis for targeted national discussion within an evolving international INR field. Anchored in real-world clinical practice and informed by the broader international context, these data support coordinated initiatives aimed at improving consistency, sustainability and patient safety.

## Supplementary information

ELECTRONIC SUPPLEMENTARY MATERIAL

## Data Availability

Data generated in this study are available from the corresponding author upon reasonable request.

## Abbreviations

BSITN: Belgian Society for Interventional and Therapeutic Neuroradiology
cSDH: Chronic subdural hematoma
INR: Interventional neuroradiology
