# Supplementary material for: Organization of Interventional Neuroradiology in Belgium: results of a national survey
Source: Insights Imaging. 2026 Oct 7;17:242. doi: 10.1186/s13244-026-02416-z (PMC13646237; doi:10.1186/s13244-026-02416-z)
Supplement: Supplementary file 1 [file 13244_2026_2416_MOESM1_ESM.pdf]

Organization of Interventional Neuroradiology in Belgium: Results of a National Survey

ELECTRONIC SUPPLEMENTARY FIGURE

Supplementary Table S1 Practice organization, procedural workload and multi-center activity among Belgian interventional neuroradiologists.

Domain	Parameter	n (%)
**Individual procedural volume**	>100 procedures/year	16 (45.7)
	51–99 procedures/year	11 (31.4)
	20–50 procedures/year	7 (20.0)
	<20 procedures/year	1 (2.9)
**Procedures commonly performed**	Aneurysm coiling/remodeling	32 (91.4)
	Vasospasm treatment	31 (88.6)
	Stent-assisted coiling	29 (82.9)
	Carotid artery stenting	28 (80.0)
**Team-level volumes (>100/year)**	Mechanical thrombectomy	22/34 (64.7)
	Aneurysm embolization	14/35 (40.0)

Supplementary Table S2 Training pathways, certification status on-call organization and remuneration models.

Parameter	n (%)
**Participation in INR on-call system**	30 (85.7)
**>6 on-call shifts/month**	16 (45.7)
**Completed dedicated INR fellowship**	29 (82.9)
**Formal INR diploma/certification**	12 (34.3)
**No specific on-call compensation***	8/33 (24.2)
**Mixed system (fixed+ act-based payment) ***	10/33 (30.3)

**Percentages are calculated using the number of respondents answering each question as the denominator. Remuneration data is available for 33 respondents.*

Supplementary Table S3. Factors associated with perceived volume sufficiency among Belgian interventional neuroradiologists. Respondents were dichotomized based on their response to the question "Do you feel your volume sufficient across all neurointerventional procedures?" Comparisons were performed using Fisher's exact test. Bold indicates statistical significance (p<0.05).

**Variable**	**Satisfied _%**	**Unsatisfied _%**	**p**
**>10 years experience**	66.7	8.3	**0.001**
**High personal volume (≥51)**	79.2	66.7	0.443
**Multi-center practice**	8.3	0	0.543
**Dedicated INR team**	41.7	50	0.729
**Dedicated technicians**	50	41.7	0.732
**Fellowship completed**	87.5	66.7	0.190
**Has INR diploma**	33.3	33.3	1
**University practice**	29.2	66.7	0.071

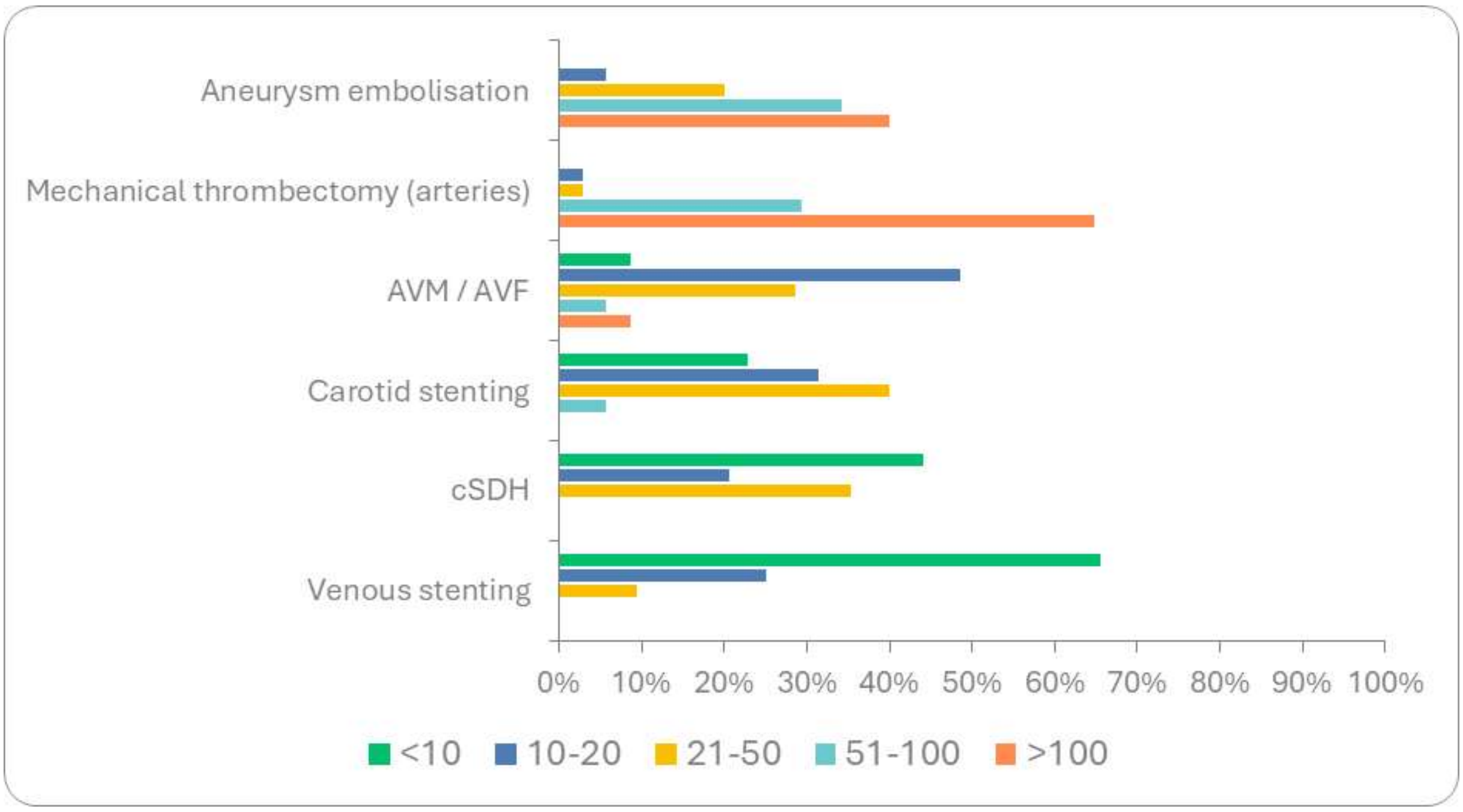

Supplementary Figure S1. Distribution of annual team-level procedural volumes by intervention, based on responses to the survey question: "What is your team's annual procedural volume for each of the following INR procedures?" Responses are shown across predefined volume categories (<10, 10–20, 21–50, 51–100 and >100 procedures per year). *Abbreviations: AVM, arteriovenous malformation; AVF, arteriovenous fistula; cSDH, chronic subdural hematoma.*
